# High-Efficient Cs_2_AgBiBr_6_ Perovskite Solar Cells with Rare-Earth-Doped Absorber and Front Contact: A Numerical Modeling in SCAPS-1D Framework

**DOI:** 10.3390/molecules31152718

**Published:** 2026-08-05

**Authors:** Eli Danladi, Daniel Thomas, Bala I. Adamu, Setumo V. Motloung, Mokhotjwa S. Dhlamini

**Affiliations:** 1 Department of Mathematical and Physical Sciences, Central University of Technology, Bloemfontein 9300, Free State, South Africa; eli.danladi@fuhso.edu.ng; 2 Department of Physics, Federal University of Health Sciences, Otukpo P.M.B 145, Benue State, Nigeria; 3 Department of Physics, College of Science, Engineering and Technology, University of South Africa, Johannesburg 1710, South Africa; 29130794@mylife.unisa.ac.za (D.T.); adamub@unisa.ac.za (B.I.A.); 4 Directorate: Postgraduate Studies, Central University of Technology, Private Bag X 20539, Bloemfontein 9300, South Africa; smotloung@cut.ac.za

**Keywords:** perovskite solar cell, SCAPS-1D, doping, hole transport layer

## Abstract

This work proposes a simplified HTL-free PSC structure based on Cs_2_AgBiBr_6_ doped with praseodymium (Pr^3+^). By reducing interfacial layers, this design reduces defect-induced recombination and increases the resistance of the device under environmental stresses (such as temperature, oxygen, and humidity). The performance of the Pr^3+^-doped PSC (Cs_2_Ag_0.95_Pr_0.05_BiBr_6_) with both FTO and Tb-FTO as front contacts were investigated using solar capacitance simulation software (SCAPS-1D) version 3.3.10. The FTO-based reference device showed photovoltaic parameters of *V*_oc_ = 0.86 V, *J*_sc_ = 12.52 mA/cm^2^, FF = 70.13%, and PCE = 7.51%, while the Tb-doped FTO device showed *V*_oc_ = 0.86 V, *J*_sc_ = 12.67 mA/cm^2^, FF = 72.98%, and PCE = 7.91%. The performance of the device was analyzed based on variation in absorber thickness and defect density, ETL thickness and dopant concentration, band gap, and ETL/absorber interface defect density in the Tb-FTO/TiO_2_/Cs_2_Ag_0.95_Pr_0.05_BiBr_6_/C to obtain optimal values of 1.0 μm, 10^13^ cm^−2^, 0.09 μm, 10^21^ cm^−2^, 1.5 eV, and 10^8^ cm^−3^. Utilizing these optimized values, the final device predicted a PCE of 19.93%, FF of 84.51%, *J*_sc_ of 27.73 mA/cm^2^, and *V*_oc_ of 0.85 V. The device was also found to be sensitive to variations in the back-contact work function, ambient temperature, series and shunt resistances. A PCE of ~27.65% was achieved at higher metal work function (e.g., *W*_F_ = 5.9 eV for Se), with a corresponding FF of ~82.69%, *J*_sc_ of ~27.78 mA/cm^2^, and *V*_oc_ of 1.20 V. Therefore, while direct experimental validation for the proposed HTL-free structure is not yet available, the comparison with experimentally demonstrated HTL-containing counterparts provides confidence in the predictive capability of our model. We expect that the present work will serve as a theoretical foundation and motivation for future experimental fabrication and characterization of HTL-free devices.

## 1. Introduction

Research into sustainable photovoltaic technologies has accelerated due to rising global energy demands and environmental issues related to fossil fuel. Perovskite photovoltaic devices have exhibited outstanding efficiency > 26% due to their superior optoelectronic characteristics, especially lead-based ones like MAPbI_3_ [1,2,3]. However, the poor stability of the material under ambient conditions and innate toxicity of lead continue to hamper their widespread commercialization [4]. This difficulty has spurred the search for alternatives that are lead-free, of which the A_2_B′B″X_6_ family of double perovskites has emerged as a strong contender for applications. Due to its exceptional structural stability, non-toxic nature, and suitability for both indoor and outdoor photovoltaic applications, Cs_2_AgBiBr_6_ has become one of the most appealing options [5,6]. Other lead-free double perovskites, such as Cs_2_AgInCl_6_, Cs_2_AgSbBr_6_, and Cs_2_NaBiCl_6_, have also gained attention. Despite their optical tunability, Cs_2_AgInCl_6_′s broad bandgap (~3.3 eV) hinders the efficient absorption of visible light. Similarly, Cs_2_NaBiCl_6_ is environmentally benign and stable but has poor charge transport [7]. Cs_2_AgSbBr_6_ shows some promise due to the narrower bandgap (~1.95–2.0 eV), but its synthesis is still challenging, with only a few reports on its device performance [2,3,8,9]. Conversely, Cs_2_AgBiBr_6_ is characterized by a bandgap of ~2.15 eV, reliable thermal durability, and simple preparation methods, which position it as a promising material and call for its use as the main absorbing material for this study [10]. Despite its promising features, there are two main issues that limits its applications, which are high defect densities that cause non-radiative recombination and a comparatively large bandgap (~2.15 eV) that limits the absorption of visible light. When considered, these factors reduce device efficiency [2,11].

Significant obstacles still exist despite using several strategies, such as charge transport layer optimization (e.g., Spiro-OMeTAD, PTAA) and heterovalent doping (e.g., Rb^+^, Sb^3+^), to enhance efficiency. Hygroscopic additives such as Li-TFSI and TBP are used in traditional doped hole transport layers (HTLs) to enhance moisture-related degradation. While dopant-free and hydrophobic HTLs such as poly(3-hexylthiophene) (P3HT) are more stable and less expensive, they also have interfacial recombination losses, which reduce the open-circuit voltage in Cs_2_AgBiBr_6_-based devices. In Cs_2_AgBiBr_6_-based PSCs, fluorine-doped-tin-oxide (FTO) substrates are frequently used due to their high transparency and chemical stability, but they also pose a number of significant issues that restrict device performance. Firstly, effective charge transport is impeded by the relatively high sheet resistance of FTO, which raises series resistance and lowers overall power conversion efficiency. Secondly, the electron transport layer (ETL), such as TiO_2_ or SnO_2_ and FTO, may not align their energy levels well, which could create a barrier at the interface that would hinder electron extraction and encourage interfacial charge recombination [3]. The morphology and crystallization of the Cs_2_AgBiBr_6_ film can also be adversely affected by the surface roughness and non-uniformity of the FTO layer, which can result in defects and inadequate film coverage. FTO may also experience thermal and chemical degradation, changing its optical and conductivity properties during high-temperature or prolonged processing conditions. In Cs_2_AgBiBr_6_ double-perovskite solar cells, these combined problems lead to less charge extraction, higher recombination losses, and decreased device stability. Importantly, no previous study has simultaneously increased stability and efficiency by combining rare-earth-based doping of Cs_2_AgBiBr_6_ and FTO.

To overcome these constraints, we suggest a triple-modification strategy in our proposed Cs_2_AgBiBr_6_-based device as follows: (1) using a praseodymium (Pr^3+^)-doped Cs_2_AgBiBr_6_ material with reduced bandgap and improved charge carrier mobility, (2) eliminating HTL to have an HTL-free device with a lower number of interfaces that serve as recombination centers, and (3) using terbium (Tb)-doped FTO, which is known to have improved electrical conductivity and alter surface electronic properties [12,13]. Pr^3+^ was chosen as a dopant due to its suitable ionic radius and oxidation state, which enable it to be placed efficiently within the Cs_2_AgBiBr_6_ lattice while preserving the structural formation. Pr^3+^ exhibits superior ability to simultaneously tune the electronic structure, reduce defect density, and improve charge transport properties compared to other rare-earth and transition-metal dopants. Prior research has shown that by creating intermediate electronic states and preventing the formation of halide vacancies, Pr^3+^ incorporation can greatly enhance light-harvesting ability and reduce non-radiative recombination [8,14,15]. Pr^3+^ has an optimized balance state between band structure modification and defect passivation, making it a reliable option for improving the optoelectronic performance of Cs_2_AgBiBr_6_-based perovskites. Replacing Sn^4+^ ions with Tb^3+^ increases the number of charge carriers (electrons) to the FTO lattice, which increases carrier mobility and concentration. Additionally, doping with Tb can slightly adjust the work function, improve energy level alignment and lower interfacial recombination losses.

Therefore, in this research work, we improve the charge carrier transport and raise the overall photovoltaic efficiency of Cs_2_AgBiBr_6_-based solar cells through the utilization of a Pr^3+^-doped absorber layer and Tb-doped FTO by device simulation using solar capacitance simulation software (SCAPS-1D). The SCAPS-1D is used to solve the Poisson and continuity equations under light and applied bias to model band energy properties, charge recombination, interface, current–voltage, defect effects and other device properties. Our findings reveal that this approach predicts a record PCE of 19.93%, which is considered among the highest values documented for HTL-free doped Cs_2_AgBiBr_6_ perovskite solar cells and underscore its potential for developing stable, high-performance, and eco-friendly photovoltaic devices.

## 2. Results and Discussion

### 2.1. Validation of Measurement with FTO and Tb-FTO

**Figure 1:**
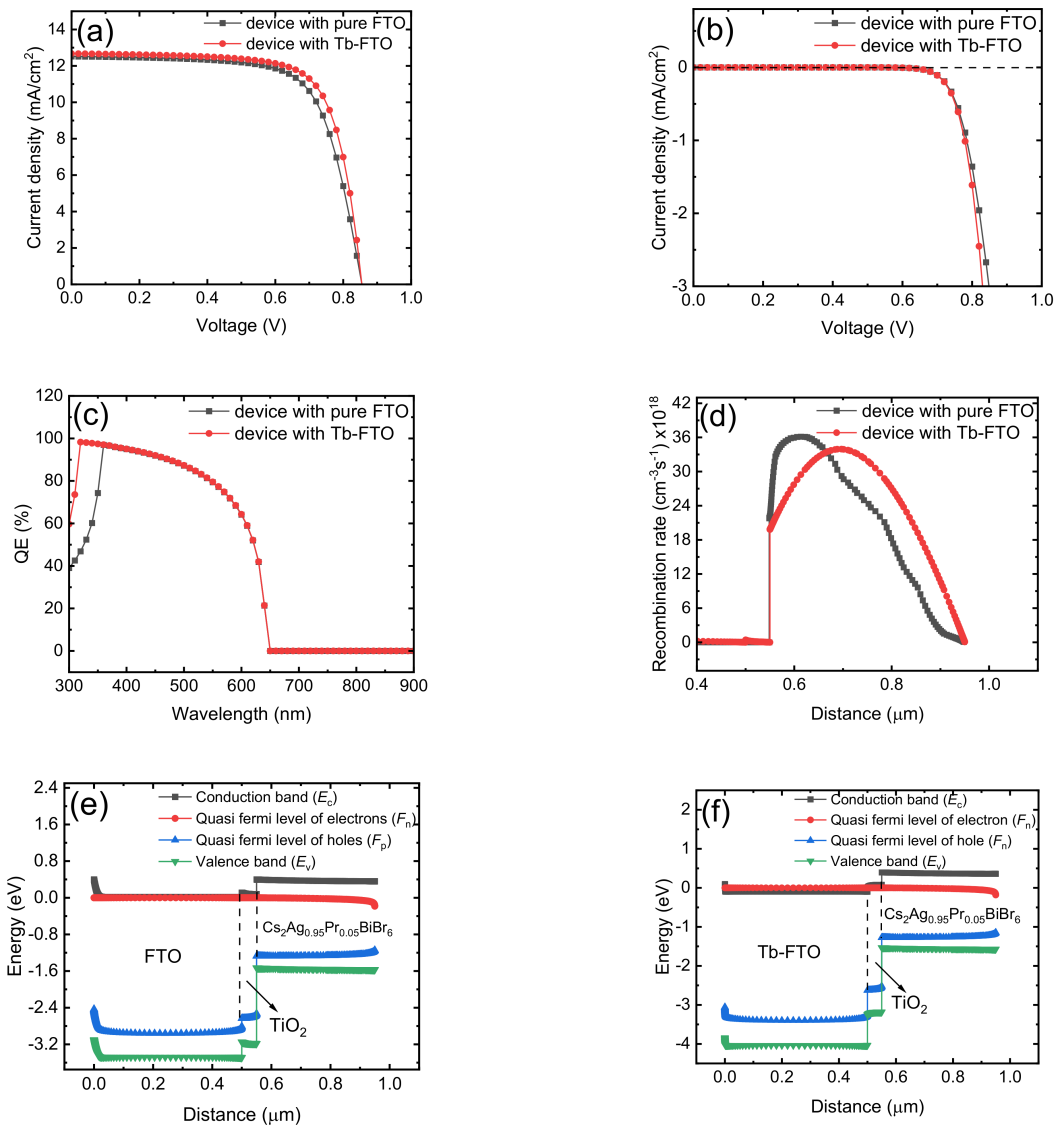
(**a**) *J-V* curve under light, (**b**) *J-V* curve in the dark, (**c**) QE curve, (**d**) recombination profile, (**e**) energy diagram for pure FTO, and (**f**) energy diagram for Tb-FTO.

The effects of using FTO and Tb-FTO as front contacts in a Cs_2_Ag_0.95_Pr_0.05_BiBr_6_ perovskite solar cell were examined by simulating the devices in both light and dark conditions. A graph of current–voltage data was generated, as shown in Figure 1a,b. Figure 1c shows the quantum efficiency, Figure 1d shows the recombination profile, and Figure 1e,f show their energy band diagrams. The study by Ullah et al. [2] was used to calibrate the model and validate the simulation results. A comparison of simulation outcomes and experimental data is presented in Table 1, with particular focus on important photovoltaic parameters like PCE, FF, *J*_sc_, and *V*_oc_. The results for the device using FTO were *V*_oc_ = 0.86 V, *J*_sc_ = 12.52 mA/cm^2^, FF = 70.13%, and PCE = 7.51%. The results changed to *V*_oc_ = 0.86 V, *J*_sc_ = 12.67 mA/cm^2^, FF = 72.98%, and PCE = 7.91% when Tb-FTO was used. Device performance is better for the Tb-FTO configuration than for the pure FTO counterpart. This remarkable performance is ascribed to the distinctive optoelectronic behavior brought about by terbium (Tb) doping. Localized 4f orbital states are introduced by the interaction of the rare-earth metal Tb with the electronic structure when it is integrated into the FTO matrix. This modification improves electrical conductivity by raising the concentration of free carriers in the FTO layer [12,13,16].

Additionally, Tb doping improves the overall quality of the interface by suppressing trap-assisted recombination through passivating oxygen vacancies and other intrinsic defects in the transparent conducting oxide (TCO). Tb also increases photoexcitation and broadens absorption of the solar spectrum by permitting sub-bandgap photon upconversion and enhancing photon management through improved optical scattering [13]. In addition to promoting effective electron transfer into the TCO, the optimized band alignment between the ETL and the Tb-doped FTO lowers interfacial recombination losses. A strong internal electric field and adequate carrier diffusion lengths are indicated by the higher *J*_sc_, which permits effective charge extraction. This effect is further controlled by the elimination of the organic spiro-OMeTAD, which reduces number of interfaces and facilitates better charge transport by offering fewer boundaries for recombination [17]. The device using undoped FTO, on the other hand, performs somewhat worse, with an overall efficiency of 7.51%. The performance degradation can be attributed to increased resistive losses and suboptimal band alignment at the ETL/FTO interface, which hinder efficient charge extraction and enhance recombination losses [13]. Due to the improved light absorption and enhanced charge extraction capability of the Tb-doped FTO device, it exhibits a more uniform and extended response across the wavelength range in terms of quantum efficiency (QE) characteristics (see Figure 1c). The QE profile, which reflects the device’s spectral performance, is a crucial indicator of its photon-to-current conversion efficiency. The device employing undoped FTO, on the other hand, typically exhibits lower quantum efficiency at shorter wavelengths, which is consistent with its reduced *J*_sc_ values and limited carrier mobility. The recombination rate is more pronounced in the device without Tb, with its value attaining a magnitude of 36 × 10^18^ cm^−3^s^−1^ (see Figure 1d).

**Table 1:** *J-V* parameters of simulated and experimental results.

Device	Study	*V*_oc_	*J*_sc_	FF	PCE	Remark
FTO/TiO_2_/Mo-Cs_2_AgBiBr_6_/Spiro-OMeTAD/Au	Exp.	0.94	5.59	67.00	3.95	[18]
FTO/TiO_2_/Cs_1.96_li_0.01_Na_0.03_AgBiBr_6_/C	Exp.	1.07	6.56	71.60	5.02	[19]
ITO/SnO_2_/Cs_2_AgBiBr_6_/spiro-OMeTAD/Au	Exp.	0.92	11.4	60.93	6.37	[20]
FTO/Ti/ZTO-Cs_2_Ag_0.95_Mg_0.05_BiBr_6_/Au	Exp.	0.90	5.77	76.00	3.98	[21]
FTO/TiO_2_/Cs_2_Ag_0.95_Pr_0.05_BiBr_6_/P3HT/Spiro-OMeTAD	Exp.	0.90	5.01	81.1	3.88	[2]
FTO/TiO_2_/Cs_2_Ag_0.95_Pr_0.05_BiBr_6_/Au	Sim.	0.86	12.52	70.13	7.51	[This work]
Tb-FTO/TiO_2_/Cs_2_Ag_0.95_Pr_0.05_BiBr_6_/Au	Sim.	0.86	12.67	72.98	7.91	[This work]

When the energy levels of two contacting materials are aligned, an equilibrium is created that controls the movement of electrons and holes across an interface [22]. This process is described by band alignment, which is essential to the functioning of electronic and optoelectronic devices like solar cells and transistors [23]. Band alignment is classified into either Type I (cliff) or Type II (spike) [23,24], which has a distinct effect on carrier transport. In Type I band alignment, electron transfer is favored from higher to lower energy states, where the valence band maximum of one material lies below or aligns with the conduction band minimum of the adjacent material.

When light is applied to the device, two distinct quasi-Fermi levels are established for electrons (*F*_n_) and holes (*F*_p_). Their formation indicates the creation of electron–hole pairs within the solar cell. Figure 1e,f show that the hole *F*_p_ occupies the region between *E*_v_ and its quasi-Fermi level, while the electron *F*_n_ lies between *E*_c_ and the corresponding quasi-Fermi level. Key parameters like the built-in potential, internal electric field, and interfacial resistance at the Cs_2_Ag_0.95_Pr_0.05_BiBr_6_/ETL/Tb-FTO and Cs_2_Ag_0.95_Pr_0.05_BiBr_6_/ETL/FTO interfaces are influenced by slight offsets in the *E*_c_ and *E*_v_. The horizontal axis (abscissa) in the energy band diagram indicates distance in micrometers (µm), and the vertical axis (ordinate) represents energy levels in electron volts (eV). The relative positioning of the energy band of CBO at the Tb-FTO/ETL/Cs_2_Ag_0.95_Pr_0.05_BiBr_6_ or Cs_2_Ag_0.95_Pr_0.05_BiBr_6_/ETL/FTO junctions has a significant impact on solar cell efficiency. According to Srivastava, Kasliwal, & Shirage, [25], better device performance is usually linked to ideal band offsets. According to our simulation results, the Tb-FTO/ETL/Cs_2_Ag_0.95_Pr_0.05_BiBr_6_ configuration has a favorable spike-type alignment and is therefore more efficient than the Cs_2_Ag_0.95_Pr_0.05_BiBr_6_/ETL/FTO-based structure, which has a cliff-type alignment and performs worse. According to the simulation, the quasi-Fermi level lies near the center between the VB and CB. Furthermore, recombination losses are more substantial in the FTO counterpart, while the Tb-FTO device exhibits a higher carrier generation rate.

### 2.2. Impact of Varying Defect Density (N_t_) and Absorber Thickness

One important factor affecting solar cell performance is the perovskite layer’s thickness. In general, increasing this thickness encourages greater charge carrier generation and improves light absorption [17]. Experimental and simulation studies are often performed to investigate the influence of various layer properties, with the aim of identifying optimal thicknesses and highlighting the importance of systematic optimization. Similar to this, the absorber *N*_t_ is crucial for determining the efficiency of charge extraction and recombination, so choosing it carefully is crucial to attaining the best device performance. During doping processes or device fabrication, various defects can be formed in the solar cell, including vacancies, interstitials, Schottky, and Frenkel defects, which can be found at the surface of the material or within it [26,27]. In addition, structural defects such as dislocations and grain boundaries are commonly observed in the absorber [28], and self-doping can introduce unintended impurities, which may promote the formation of additional defect states [29]. These defects produce trap states that make it easier for charge carriers to recombine non-radiatively, which lowers device efficiency. The overall performance of PSCs is largely dependent on the defect density in the absorber [3].

**Figure 2:**
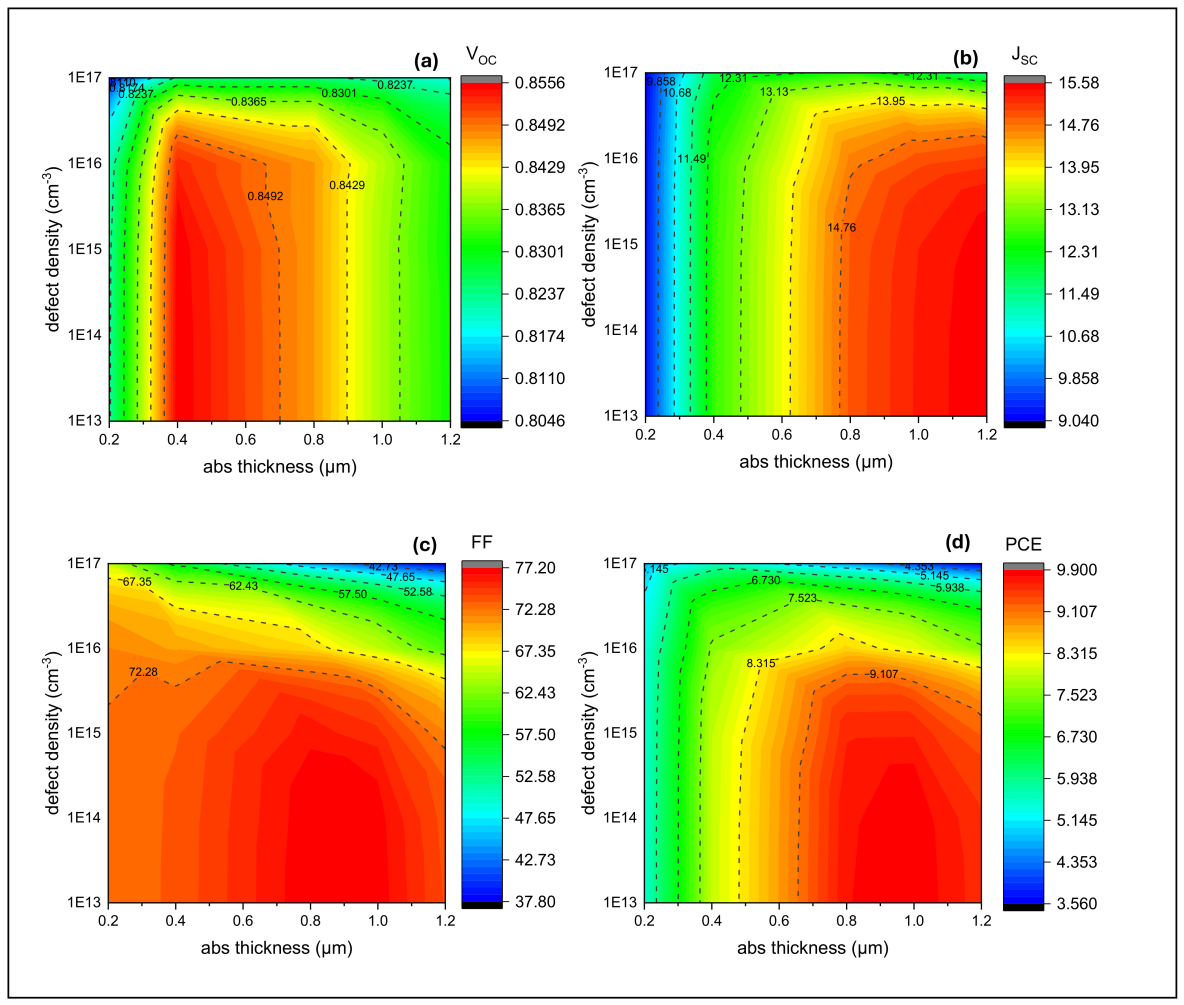
Contour plots of the PSC illustrating (**a**) *V*_oc_, (**b**) *J*_sc_, (**c**) FF, and (**d**) PCE as a function of absorber thickness and defect density.

Figure 2 presents the contour mapping of the solar cell performance as a function of simultaneous variations in defect density and absorber thickness across the investigated ranges of 10^13^–10^17^ cm^−2^ and 0.2–1.2 μm. Different values of PCE, FF, *J*_sc_, and *V*_oc_ were obtained across the examined thickness and defect ranges. Figure 2a–d illustrate the dependence of these performance metrics on absorber thickness and defect density. The analysis employed 25 iterative points per metric to enhance the accuracy of the optimization process.

The *V*_oc_ is somewhat sensitive to the thickness and defect density, as seen in Figure 2a. With thickness values ranging from 0.2 to 1.2 μm and the *N*_t_ varying from 10^13^ to 10^17^ cm^−2^, the *V*_oc_ falls within the range of 0.80–0.86 V across the grid, with a maximum *V*_oc_ = ~0.8555 V, which occurs at 0.4 μm thickness and 1 × 10^13^ cm^−2^ defect. Depending on the degree of defect, *V*_oc_ gradually decreases for thicker films (0.8–1.2 µm) after increasing slightly from 0.2 to 0.4 µm, indicating improved charge collection at moderate thickness. *V*_oc_ decreases by ~0.03 to 0.05 V in the dataset when the *N*_t_ increases from 10^13^ to 10^17^ cm^−2^. In general, there is a weakly negative correlation between *V*_oc_ and thickness and defect density [3]. *V*_oc_ decreases as defect density rises. *V*_oc_ is particularly susceptible to splitting and recombination at the quasi-Fermi level. This is in accordance with the conventional hypothesis that a higher defect/trap density leads to more nonradiative recombination, which lowers *V*_oc_ [17].

The dependence of *J*_sc_ on absorber layer thickness and doping concentration is presented in Figure 2b. To study the effects on the photovoltaic performance, thickness was varied from 0.2 to 1.2 μm, and *N*_t_ was varied concurrently from 10^13^–10^17^ cm^−2^. The maximum *J*_sc_ is 15.34 mA/cm^2^ at 1.0 μm thickness and 1 × 10^13^ cm^−3^ defect. *J*_sc_ decreases with very high defect density, which means carrier recombination lowers collection current, but it increases significantly with absorber thickness up to ~0.8 to 1.0 µm (better absorption and charge generation). Increasing the defect density from 10^13^ to 10^17^ reduces *J*_sc_ (losses from bulk recombination) for a fixed thickness. At greater thicknesses, where carriers travel farther, the effect is more noticeable. The *J*_sc_ benefits from improved injection/collection (higher effective doping/conductivity) and light harvesting (thicker absorber).

Figure 2c illustrates the variation in fill factor (FF) with increasing absorber thickness and defect density. FF varies between 38 and 77%. At the highest defect density (1 × 10^17^ cm^−3^), the FF is strongly reduced, yielding FF = ~38%. Recombination and series/parallel loss mechanisms have a strong effect on FF; bulk defect states increase recombination and cause non-ideal diode behavior or apparent series resistance, both of which sharply reduce FF. In accordance with the *J*_sc_ trend, the best FF is found near the low-defect region (77.1% at defect = 1 × 10^13^ cm^−2^ and thickness = 0.8 μm).

The dependence of PCE on absorber layer thickness and doping concentration is presented in Figure 2d. Thickness = 1.0 μm and defect density = 1 × 10^13^ cm^−2^ yield the highest efficiency of approximately 9.891%, while thickness = 1.2 μm and defect density = 1 × 10^17^ cm^−3^ yield the lowest efficiency of ~3.566%. The combined effect of *V*_oc_, *J*_sc_, and FF is the efficiency.

### 2.3. Impact of Varying Doping Concentration and ETL Thickness

The ETL, located at the interface between the absorber and FTO, is vital for device efficiency because it has a significant impact on photon interaction within the absorber. For the device to operate efficiently, ETL properties must be optimized. This study systematically examined the effects of donor concentration and ETL thickness on the performance of a Cs_2_Ag_0.95_Pr_0.05_BiBr_6_ Tb-doped FTO perovskite solar cell. To examine their combined effect on the photovoltaic metrics of the device, the donor density was varied between 10^13^ and 10^21^ cm^−3^ using a step of one order of magnitude (10^1^ cm^−3^ in log-scale representation), which is a standard approach in SCAPS-1D studies. This captures the full effect of doping concentration on device performance [30]. This logarithmic variation allows for a physically realistic representation of doping-dependent electrical properties, where carrier concentration and conductivity change exponentially rather than linearly in steps of 10^2^ cm^−3^ and the thickness of ETL was controlled between 0.01 and 0.09 μm in steps of 0.02 μm.

**Figure 3:**
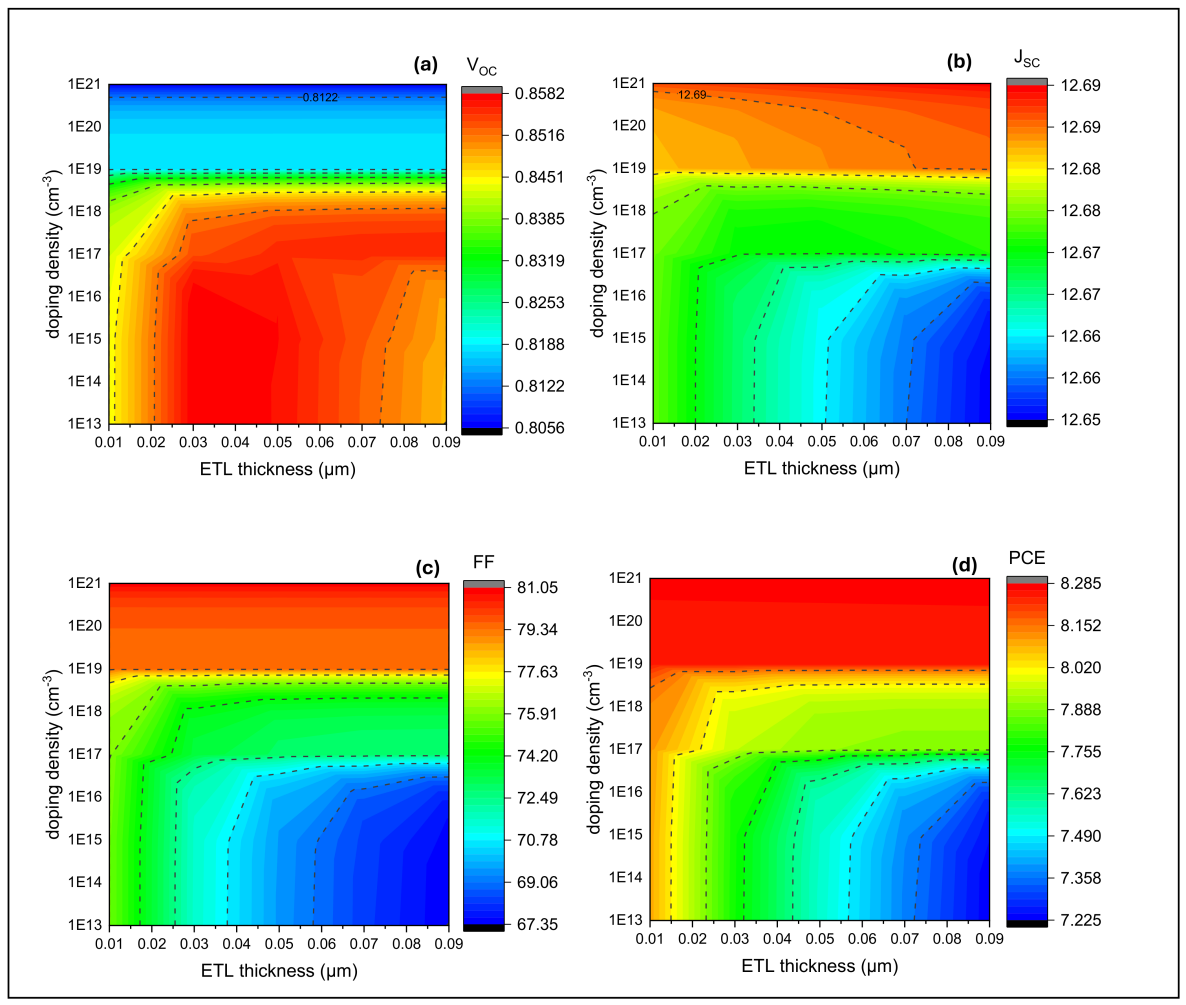
Contour mapping of the perovskite device showing (**a**) *V*_oc_, (**b**) *J*_sc_, (**c**) FF, and (**d**) PCE on the ETL thickness and *N*_D_.

Figure 3a illustrates the simultaneous effect of variations in donor density (*N*_D_) and ETL thickness on the *V*_oc_ of the PSC. The contour plot shows only a slight variation in *V*_oc_, ranging from 0.8057 to 0.8581 V. At a thickness of 0.03 µm and a doping concentration of 1 × 10^13^ cm^−3^, the maximum *V*_oc_ is ~0.8581 V. The *V*_oc_ variations are primarily attributed to recombination processes and the inherent potential of the solar cell. Optimization of the ETL thickness and *N*_D_ minimizes interfacial recombination, resulting in higher *V*_oc_. Increasing the ETL doping can enhance conductivity and carrier extraction, but it can also decrease the quasi-Fermi-level splitting observed by increasing interface recombination or shifting band alignments. In this dataset, *V*_oc_ favors lower ETL doping.

As seen in Figure 3b, changes in ETL thickness and *N*_D_ have an impact on *J*_sc_. The maximum *J*_sc_ value of ~12.693 mA/cm^2^ is achieved at 0.09 µm thickness and 1 × 10^21^ cm^−3^ doping density. The *J*_sc_ ranges from 12.654 to 12.693 mA/cm^2^. Increasing the doping results in a slight but noticeable increase in *J*_sc_ for any fixed thickness. By improving electrical conductivity and carrier extraction, increasing ETL doping lowers series resistance and enhances collection, which increases the contribution of photogenerated carriers to the measured current. The decrease in the *J*_sc_ at higher ETL *N*_D_ and lower ETL thickness is due to an increase in charge transport losses and recombination [22]. A very thin ETL reduces charge collection by increasing interfacial recombination and allowing holes to flow due to its poor electron selectivity and insufficient coverage. High doping levels in the ETL lead to significant band bending and the formation of defect-mediated recombination channels at the interface.

Figure 3c shows the changes in FF as ETL thickness and *N*_D_ are varied. The maximum FF is ~81.01% at 0.01 µm thickness and 1 × 10^21^ cm^−3^ doping concentration (very thin ETL and very high doping). The FF ranges from 67.36 to 81.01%. FF decreases with increasing ETL thickness (for the same doping level) and at low ETL doping. The high FF is the result of improved contact/selective extraction and decreased resistive losses caused by high ETL doping [30].

Figure 3d shows the contour plot of the PCE variation with respect to the variations in ETL thickness and ETL *N*_D_. The best PCE of 8.2847% is observed at ETL thickness = 0.09 µm and ETL doping = 1 × 10^21^ cm^−3^. A similar trend has been reported in the literature [17]. At the same thickness (0.09 µm) but with the lowest doping (1 × 10^13^ cm^−3^), the efficiency is lowest. This suggests that ETL doping improves PCE more than thickness does. High doping yields the best overall efficiency. The doping dominates the net effect while thickness modulates *J*_sc_ and FF. Both carrier extraction and electrical behavior are enhanced by increasing ETL doping. At the highest level of doping, *V*_oc_ is slightly decreased, but the combined gains in *J*_sc_ and FF outweigh the *V*_oc_ loss, resulting in higher PCE. The results in our simulation are opposite what is observed in a practical solar cell, which can be seen to arise from the idealized assumptions used in the SCAPS-1D model, where the ETL is treated as a defect-free, uniformly conductive layer without parasitic absorption or significant resistive losses. As a result, it enhances charge selectivity, reduces interfacial recombination, and helps capture more photons [30].

### 2.4. Effect of ETL/Absorber Defect Density (N_t_)

One of the primary sources of localized defect states is the discontinuities that occur at the interfaces between different layers of the solar cell structure [17]. Since the extraction and movement of charge carriers rely heavily on appropriate band alignment across the layers, the *E*_c_ and *E*_v_ edges were analyzed to find charge transport layers that accurately reflect the impact of band offsets on photovoltaic efficiency.

**Figure 4:**
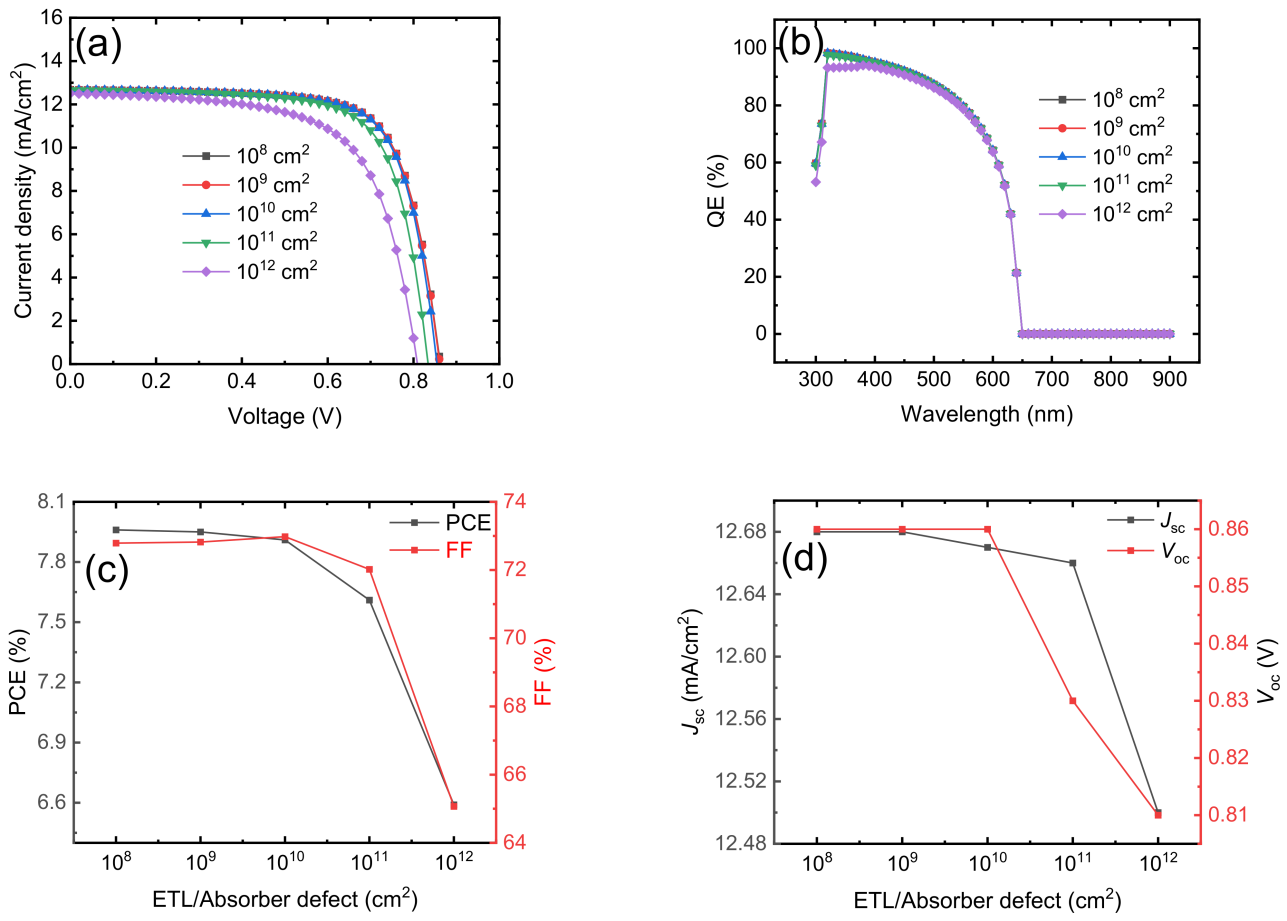
(**a**) The JV, (**b**) QE, and (**c**) PCE and FF correlation and (**d**) *J*_sc_ and *V*_oc_ correlation with ETL/absorber *N*_t_.

Keeping all other simulation parameters constant, the interface defect density (*N*_t_) was varied from 10^8^ to 10^12^ cm^−2^ to investigate its impact on device performance due to defects at the ETL/absorber interface [31]. Figure 4a displays the *J–V* characteristics for different values of interface defects, and Figure 4b displays the corresponding QE spectra. Figure 4c displays the PCE and FF trends, and Figure 4d displays the relationships between *J*_sc_ and *V*_oc_ with varying interface defects. The quantum efficiency (QE) spectrum covers the visible region of the electromagnetic spectrum. For *N*_t_ values between 10^8^ and 10^10^ cm^−2^, the FF increases and the *V*_oc_ remains constant, while beyond those values, there is a decline. This is because a moderate level of defects can enhance charge transfer across the interface. These shallow defect states can improve current flow and minimize recombination by lowering resistance and facilitating electron extraction [17]. Consequently, the device functions more effectively, resulting in a higher FF. On the other hand, an excessively high defect density leads to increased recombination, causing the fill factor (FF) to decline. As observed, both the efficiency and current density of the perovskite solar cell decrease with increasing ETL/absorber defect density, which is attributed to the formation of additional interfacial trap states at higher defect levels [3]. Due to the increased defect states, charge carriers are more likely to reach the electrodes, where they undergo recombination as trap states capture and localize the carriers. Both the overall PCE and *J*_sc_ are decreased by this loss of charges. This indicates that higher defect densities lead to increased charge carrier losses, resulting in degraded device performance. Consequently, non-ohmic contacts are formed, hindering electron–hole transport and leading to additional voltage losses in *V*_oc_ [17]. According to Table 2, the optimal device performance is attained at an ETL/absorber interface defect density of 10^8^ cm^−2^, which corresponds to a PCE of 7.96%, FF of 72.79%, *J*_sc_ of 12.68 mA/cm^2^, and *V*_oc_ of 0.86 V.

### 2.5. The Impact of the Absorber Layer Band Gap

The absorber band gap is a crucial parameter that influences optical absorption and charge carrier dynamics and, consequently, plays a decisive role in the performance of perovskite solar cells (PSCs). The study investigating the influence of the band gap on PSC performance was carried out using SCAPS-1D simulations, in which the absorber band gap was systematically varied from 1.5 to 2.1 eV. The choice of band gap was based on previously published values between 1.4 and 2.19 eV [3,32,33,34,35].

**Figure 5:**
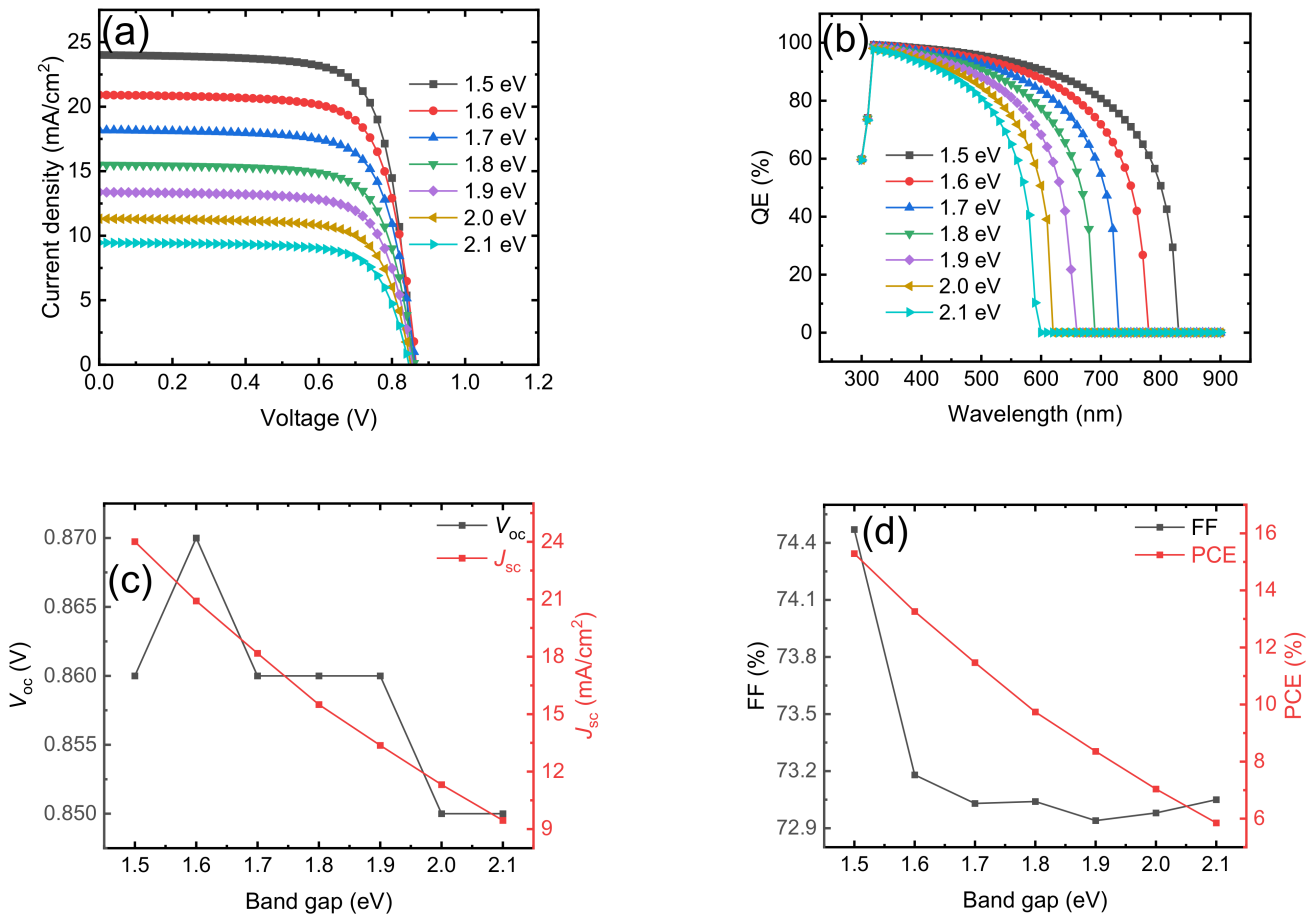
(**a**) *J-V*, (**b**) QE, and (**c**) the correlation between *V*_oc_ and *J*_sc_ and (**d**) PCE and FF at different band gaps.

The *J-V* properties of the various values of the band gap are presented in Figure 5a. Some of the factors that determine the shape of the J–V curve include intrinsic material properties, device architecture, illumination conditions, and operating temperature [36]. Based on these characteristics, basic photovoltaic metrics, i.e., the *J*_sc_, *V*_oc_, FF, and PCE, were determined. The findings show that the band-gap variation has a significant impact on all performance metrics, justifying the relevance of the band-gap optimization in the design of the perovskite solar cell. The increase in the absorber’s band gap from 1.5 to 2.1 eV results in a significant decrease in *J*_sc_ and PCE. This can be explained by the fact that it lowers the absorption of photons and the generation of charge carriers. The narrower the bandgap, the less the absorption with photons of lower energy that are absorbed at higher energies and transmitted without contributing to photogeneration. Therefore, the decreased concentration of carriers results in a considerable reduction in the density of the current and overall efficiency of the device. On the other hand, the *V*_oc_ and FF variations as a function of increasing band gap exhibit nonlinear characteristics as a result of the intricate interactions between charge transportation, recombination processes, and resistance [37]. At band gap values between 1.5 and 1.7 eV, an augmentation in photocurrent improves the impact of series resistance, which causes a limited decline in FF. As the band gap increases, the lower carrier generation decreases the current density with the reduction in resistive loss, and FF is enhanced. However, in wide-band-gap absorbers (above 1.7 eV), interfacial energy-level mismatch, greater defect densities and greater non-radiative recombination are commonly experienced [36,38]. These impacts negatively affect extraction of charges and maximize recombination losses, resulting in poor FF, as shown in Table 3. The variation in fill factor (FF) across different band gaps arises from the competing effects of resistive losses and recombination losses.

Figure 5b shows the QE spectra of the various band gaps. As the band gap increases between 1.5 and 2.1 eV, the QE gradually decreases. When the band gap is lower, the absorber is capable of absorbing photons with a wide spectral range, including visible and near infrared, and has a high quantum efficiency. The larger the band gap, the shorter the wavelength range over which it can absorb photons, meaning that low-energy photons are not absorbed and therefore do not contribute to charge carrier generation [17]. This effect is further amplified by the increase in defect density and the associated rise in non-radiative recombination, which is commonly observed in wide-band-gap perovskites. As a result, the fraction of absorbed photons contributing to photocurrent generation decreases, leading to a gradual reduction in QE. Figure 5c and Figure 5d illustrate the correlations between *V*_oc_ and *J*_sc_, and between FF and PCE, respectively.

### 2.6. Optimized Device

**Figure 6:**
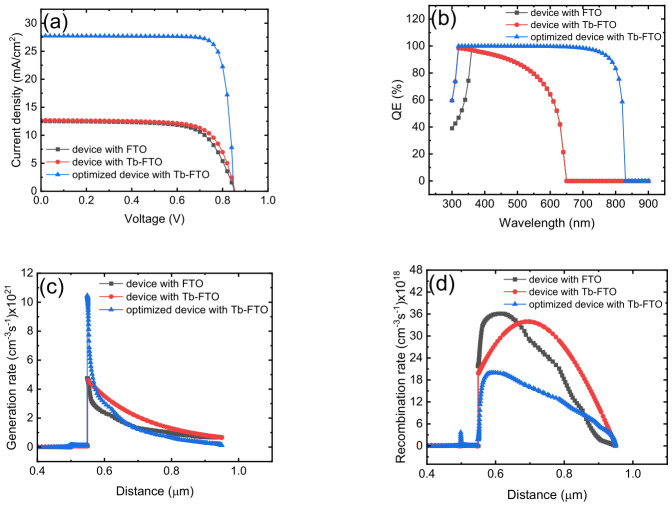
Comparison of unoptimized and optimized devices, illustrating (**a**) *J–V* characteristics, (**b**) quantum efficiency, (**c**) generation rate, and (**d**) recombination rate.

After a detailed evaluation of the individual layer parameters, the optimized values were selected and used for subsequent simulation studies. The absorber layer’s optimal thickness and defect density were found to be 1.0 μm and 10^13^ cm^−2^, respectively. Similarly, the ETL was optimized with a thickness of 0.09 μm and a doping concentration of 10^21^ cm^−3^. The optimized ETL/absorber defect density was 10^8^ cm^−2^, and the band gap was optimized to be 1.5 eV. The simulated device’s FF was 84.51%, its *V*_oc_ was 0.85 V, its maximum PCE was 19.93%, and its *J*_sc_ was 27.73 mA/cm^2^. The photovoltaic and quantum efficiency performance of the optimized versus unoptimized devices is shown in Figure 6a,b, respectively.

A comparative QE study of optimized versus unoptimized devices (Figure 6b) reveals that Tb doping and optimization extend the photoresponse range to 310–830 nm, while the unoptimized device without doping is restricted to 350–650 nm, indicating lower photon harvesting efficiency. Compared to the unoptimized device, enhanced photon-to-carrier conversion is observed in the optimized device, resulting in improved photoresponse, whereas the unoptimized device is limited by stronger recombination processes (see Figure 6c,d).

### 2.7. Evaluation of the Current SCAPS-1D Results Relative to Prior Published Results

Table 4 compares the present simulation results with previously reported data. The experimental values are consistently lower than those obtained from simulations. Previous simulation studies have also shown that achieving comparable performance metrics in lead-free perovskite solar cells requires considerable effort. Cs_2_AgBiBr_6_-based perovskite solar cells have so far demonstrated notable performance values, including a PCE of 6.37%, FF of 60.93%, *J*_sc_ of 11.4 mA/cm^2^, and *V*_oc_ of 0.92 V. After employing praseodymium (Pr^3+^)-doped Cs_2_AgBiBr_6_ with enhanced charge carrier mobility and fewer interfacial recombination centers, the simulated device exhibits optimized performance, with a PCE of 19.93%, FF of 84.51%, *J*_sc_ of 27.73 mA/cm^2^, and *V*_oc_ of 0.85 V in the configuration Tb-FTO/TiO_2_/Cs_2_Ag_0.95_Pr_0.05_BiBr_6_/Au.

### 2.8. Impact of Metal Back Contact on the Optimized Device with Varying Work Functions

**Figure 7:**
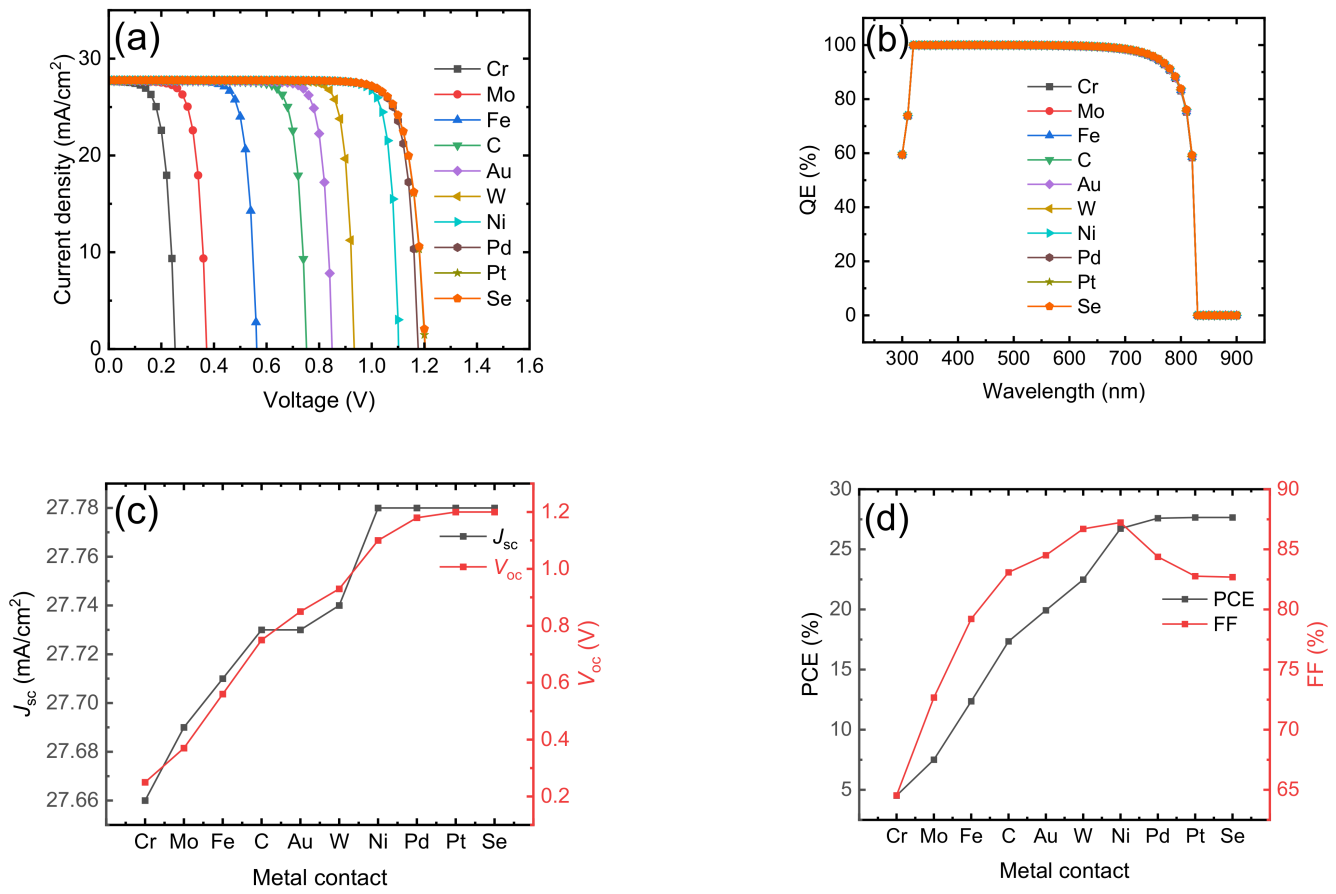
(**a**) *J-V* characteristics, (**b**) QE curve, (**c**) variation in *J*_sc_ and *V*_oc_, and (**d**) variation in PCE and FF with different W_F_ of back contact.

The PCE of 19.93%, FF of 84.51%, *J*_sc_ of 27.73 mA/cm^2^, and *V*_oc_ of 0.85 V are all achieved by the gold-based metal back contact. Furthermore, variations in the back-contact work function (*W*_F_) result in distinct photovoltaic performance characteristics [30]. This section examines the influence of the metal back-contact work function on device performance, as summarized in Table 5 and illustrated in Figure 7. As *W*_F_ increases, the metric parameters show the following trend: Cr < Mo < Fe < C < Au < W < Ni < Pd < Pt < Se. A lower metal work function leads to poorer photovoltaic performance. In the case of the Cr back contact, the low work function facilitates electron transport but hinders efficient hole extraction, resulting in increased interfacial recombination and reduced device performance. The improved photovoltaic performance observed at higher metal work functions is attributed to the reduced Schottky barrier height at the metal/perovskite interface, which facilitates efficient hole extraction and minimizes carrier recombination losses [3]. The results obtained using Se as the back-contact material are intriguing. Se is not a conventional or widely adopted back-contact electrode in perovskite solar cells [43]; however, it was used in our study due to its excellent corrosion resistance, lower ion migration, greater chemical inertness toward halide perovskites, and high work function, which aligns well with the highest occupied molecular orbital (HOMO) of many hole transport materials [22,43].

### 2.9. Temperature’s Impact on the Optimal Device

**Figure 8:**
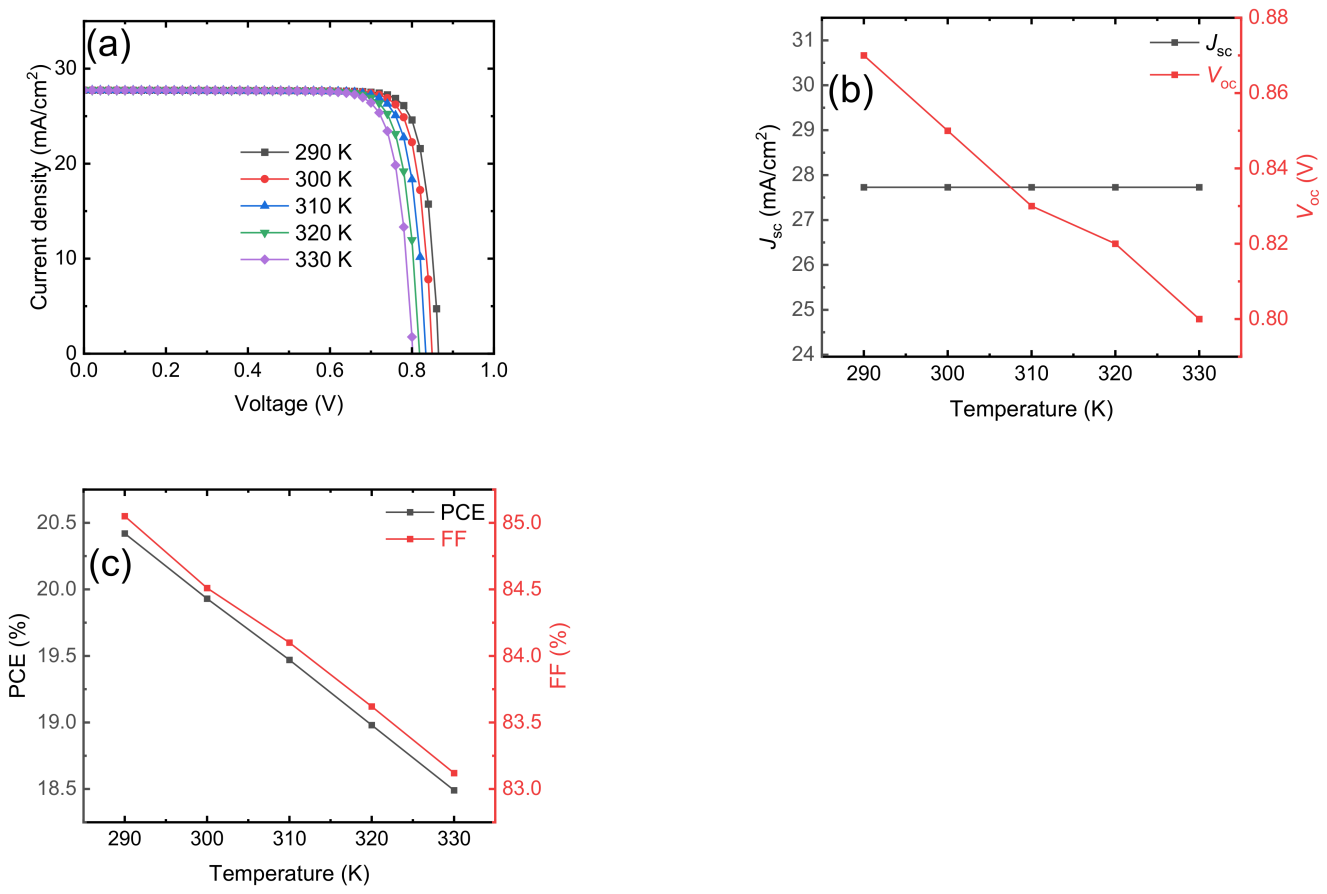
(**a**) *J–V* characteristics, (**b**) dependence of Jsc and Voc, and (**c**) trends in PCE and FF as a function of temperature.

A major obstacle to the commercialization of perovskite-based devices such as PSCs is their thermal instability under illumination, despite the excellent photon-harvesting properties of perovskite materials [44]. A photovoltaic device typically operates at a temperature (~45 °C) that is higher than ambient conditions, making temperature a critical parameter that significantly influences device performance [45,46]. Temperature fluctuations affect the lattice structure of the semiconductor, altering charge carrier mobility, electrical conductivity, and the band gap [47]. The effect of temperature variation was studied over the range of 290–330 K to evaluate its influence on the performance of the optimized PSCs. Figure 8a–c show the resulting *J–V* characteristics and the dependence between *J*_sc_ and *V*_oc_ and between PCE and FF, respectively. The graphs show that while *J*_sc_ shows constant values with increasing temperature, *V*_oc_, FF and PCE decrease as the temperature rises (see Table 6). This behavior is attributed to the generation of reverse saturation current within the PSCs, which enhances carrier recombination [17]. At elevated temperatures, increased carrier scattering reduces charge mobility, thereby raising internal resistance and lowering device performance.

### 2.10. Impact of Series Resistance on the Optimized Cell’s Performance

**Figure 9:**
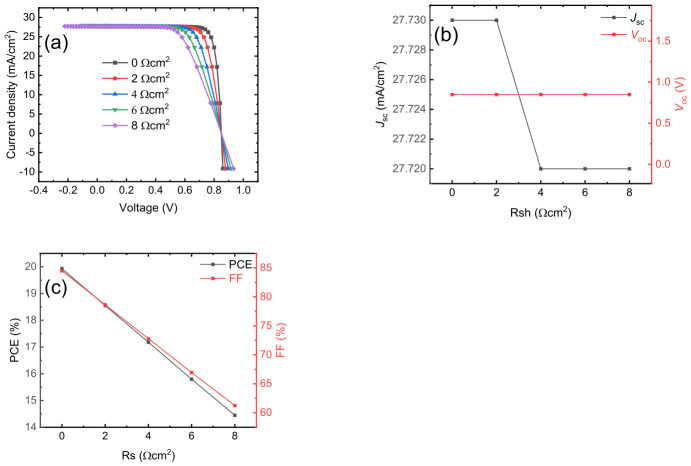
(**a**) *J–V* characteristics, (**b**) dependence of *J*_sc_ and *V*_oc_, and (**c**) trends in PCE and FF as a function of Rs.

**Figure 10:**
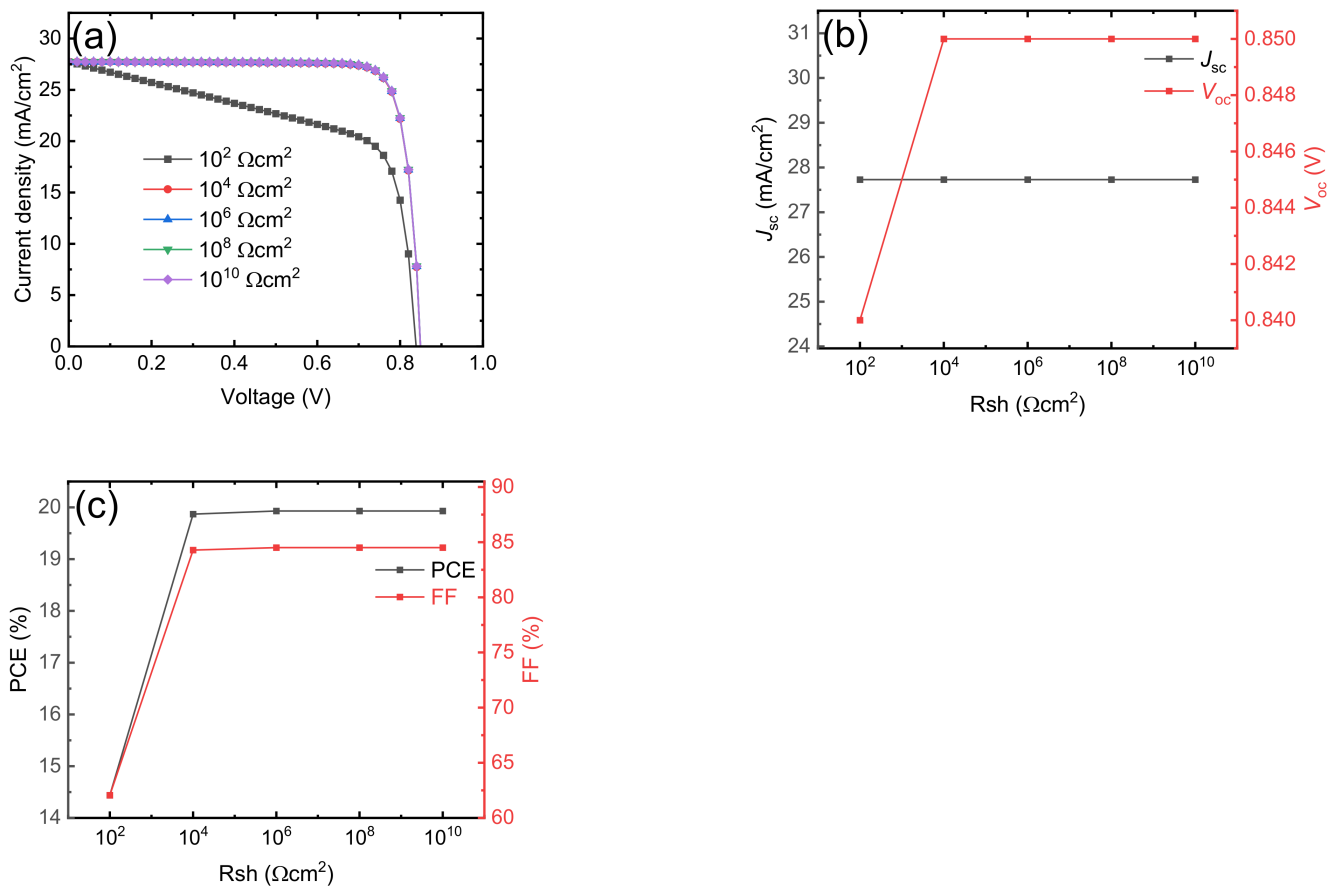
(**a**) *J–V* characteristics, (**b**) dependence of *J*_sc_ and *V*_oc_, and (**c**) trends in PCE and FF as a function of Rsh.

Three primary factors that influence the series resistance (Rs): (i) the current flowing across the absorber/HTL and ETL/absorber interfaces, (ii) the back contact/FTO interface, and (iii) the intrinsic resistance of the front and back contacts [3,48]. By doping particular layers to increase their conductivity, the Rs can be maximized [49]. By adjusting Rs from 0 to 8 Ωcm^2^ in steps of 2 Ωcm^2^, while keeping the shunt resistance (Rsh) constant at 10^5^ Ωcm^2^, the study examined the impact of Rs on the PSC’s optimized photovoltaic performance. Figure 9a shows the device’s *J–V* characteristics, while Figure 9b shows the correlation between *J*_sc_ and *V*_oc_ as a function of Rs, and Figure 10c shows the variation in PCE and FF with varying series resistance. A comparison of the outcomes for various Rs values is shown in Table 7. As Rs rises, it is clear that both PCE and FF decrease, which is in line with findings from earlier research [3]. Higher values of series resistance (Rs) increase resistive losses, thereby reducing the squareness of the *J–V* curve. Furthermore, when Rs surpasses 0 Ωcm^2^, *J*_sc_ tends to decrease slightly, most likely as a result of the decrease in transparency brought on by discoloration effects [50]. Since no current flows through the device under open-circuit conditions, variations in Rs have a negligible impact on the *V*_oc_. Since the current is zero under open-circuit conditions, the voltage drop across the series resistance (I × Rs) is also zero, meaning that no loss associated with Rs occurs at *V*_oc_. Thus, under current flow conditions, Rs primarily affects parameters like the FF and PCE, while *V*_oc_ is essentially unaffected.

### 2.11. Dependence of the Optimized Device Performance on Shunt Resistance

Current leakage and non-geminate recombination are the primary mechanisms contributing to shunt resistance in perovskite solar cells [17]. The perovskite absorber layer, interfacial barriers, charge transport layer materials, and metal back contacts are the primary contributors to Rsh [3]. Equations (1) and (2) [51] use the Shockley diode equation to describe the performance behavior of PSCs under different series resistance (Rs) conditions.

(1) $$J_{sc}=J_{PH}-J_{0}\left[exp\left(\frac{q_{e}\left(V-JR_{S}\right)}{nkT_{e}}\right)-1\right]-\frac{V-JR_{S}}{R_{sh}}$$

(2) $$V_{OC}=\left(\frac{nkT_{e}}{q_{e}}\right)ln\left\{\frac{J_{PH}}{J_{0}}\left(1-\frac{V_{OC}}{J_{PH}R_{sh}}\right)\right\}$$

In this expression, q denotes the elementary charge; JPH refers to the photocurrent density; J_0_ represents the reverse saturation current; Rs and Rsh correspond to the series and shunt resistances, respectively; n is the diode ideality factor; k is Boltzmann’s constant; and Te indicates the ambient temperature.

The *J–V* characteristics for different values of Rsh are shown in Figure 10a, the dependence of *J*_sc_ and *V*_oc_ is shown in Figure 10b, and the correlation of PCE and FF with Rsh is shown in Figure 10c. The impact of changing Rsh from 10^2^ to 10^10^ Ωcm^2^ in steps of 10^2^ Ωcm^2^ is summarized in Table 8. PCE and FF increase from 14.44 to 19.93% and from 62.06 to 84.51%, respectively, as a result of increasing Rsh. Both PCE and FF are nearly constant after Rsh values of 10^6^ Ωcm^2^. A low Rsh lowers the device’s overall efficiency by decreasing the photovoltaic output. The higher leakage currents at Rsh = 10^2^ Ωcm^2^ reduce the voltage across the external load, which lowers the FF. Furthermore, a low shunt resistance (Rsh) reduces *V*_oc_ as leakage currents reduce the potential difference across the cell terminals [3]. In contrast, *J*_sc_ remains relatively high at low Rsh, owing to enhanced light absorption and efficient initial charge carrier generation, which can be attributed to the ETL properties or device design. However, the detrimental effects of increased leakage currents on other performance metrics outweigh the associated benefits on *J*_sc_. At Rsh of 10^6^ Ωcm^2^, the maximum PCE of 19.93% is achieved.

## 3. Materials and Methods

Photovoltaics has advanced significantly, driven in part by the use of numerical tools that have supported and accelerated its development process [52]. Researchers from Ghent University, Belgium, developed the one-dimensional simulation software also known as SCAPS-1D [53]. Both science and engineering depend on modeling and simulation to develop efficient and reasonably cost-effective photovoltaic devices. Its key benefits lie in reducing computational time, providing reliable performance, and enabling optimization of device parameters [17,24,54].

The SCAPS-1D version 3.3.10 simulator enables electrical characterization of solar cells, including their *C–f* characteristics, *Q*–*E*, *C*–*V* response, and *J*–*V* behavior, for the purpose of device optimization [55]. The SCAPS-1D model serves as a detailed reference for the development of high-efficiency devices and enables the identification of optimal device configurations. A number of features have been incorporated into the simulator interface over time, including temperature variation, tunneling effects, grading, defect modeling, and interfacial recombination. These developments have improved predictive accuracy and enabled broader practical applications.

Since its original formulation, SCAPS-1D has played a significant role in clarifying the fundamental principles of photovoltaic devices and has become an important tool for supporting research in solar cell design and fabrication [53]. The software works by numerically integrating the semiconductor equations in an iterative manner and by taking into account the spatial distribution of the electron and hole densities [3]. In order to have reliable results of the simulations, the input parameters have to be carefully selected. The Poisson equation is expressed in Equation (3), with Equations (4) and (5) representing the charge carrier continuity relations [56].

(3) $$\frac{d^{2}}{{dx}^{2}}\psi \left(x\right)=\frac{q}{{Ɛ}_{0}{Ɛ}_{r}}\left[p\left(x\right)-n\left(x\right)+N_{D}^{+}-N_{A}^{-}\right]$$

(4) $$\frac{\partial n}{\partial t}=\frac{1}{q}\frac{{\partial J}_{n}}{\partial x}+\left(G_{n}-R_{n}\right)$$

(5) $$\frac{\partial p}{\partial t}=\frac{1}{q}\frac{{\partial J}_{p}}{\partial x}+\left(G_{p}-R_{p}\right)$$

where:

ψ defines the electrostatic potential, N_A_ the concentration of acceptor impurities, Ɛ_r_ the material’s relative permittivity, Ɛ_0_ the permittivity of a vacuum, N_D_ the donor impurity level, and n(x) and p(x) the densities of electrons and holes. The symbols G, R, q, J_n_, and J_p_ represent the carrier generation rate, recombination rate, elementary charge, electron current density, and hole current density, respectively.

(6) $$FF=\frac{J_{mp}V_{mp}}{J_{sc}V_{oc}}$$

(7) $$n=\frac{J_{sc}V_{oc}FF}{P_{in}}$$

where:

*J*_mp_ stands for maximum achievable current, *V*_mp_ for maximum achievable voltage, *J*_sc_ for short circuit current, and *V*_oc_ for open circuit voltage.

All simulations were conducted in the usual operating conditions of 25 °C, an illumination frequency of 1 × 10^16^ Hz and an incident light of 100 mW/cm^2^. A defect layer was utilized to incorporate the effect of charge recombination on the metric of the devices by using the interface between the ETL and the absorber. Table 9 presents the summary of the material properties of every functional layer, and Table 10 lists the characteristics of the defects at the interface. The model [57] did not consider any optical reflection at all interfaces and surfaces. The parameter optimization was carried out using a control–variable approach, where individual parameters were systematically varied while keeping others constant. Defect states within the absorber layer were represented using a Gaussian energy distribution centered at the neutrality level, with a characteristic energy width of 0.1 eV. After optimizing the CTL, the effects of absorber properties on device performance were systematically investigated using batch and contour simulations. The key factors analyzed were absorber defect density, absorber thickness, ETL thickness, ETL doping concentration, and band gap. Furthermore, the effects of metal back contact and temperature were evaluated in addition to series and shunt resistances.

**Figure 11:**
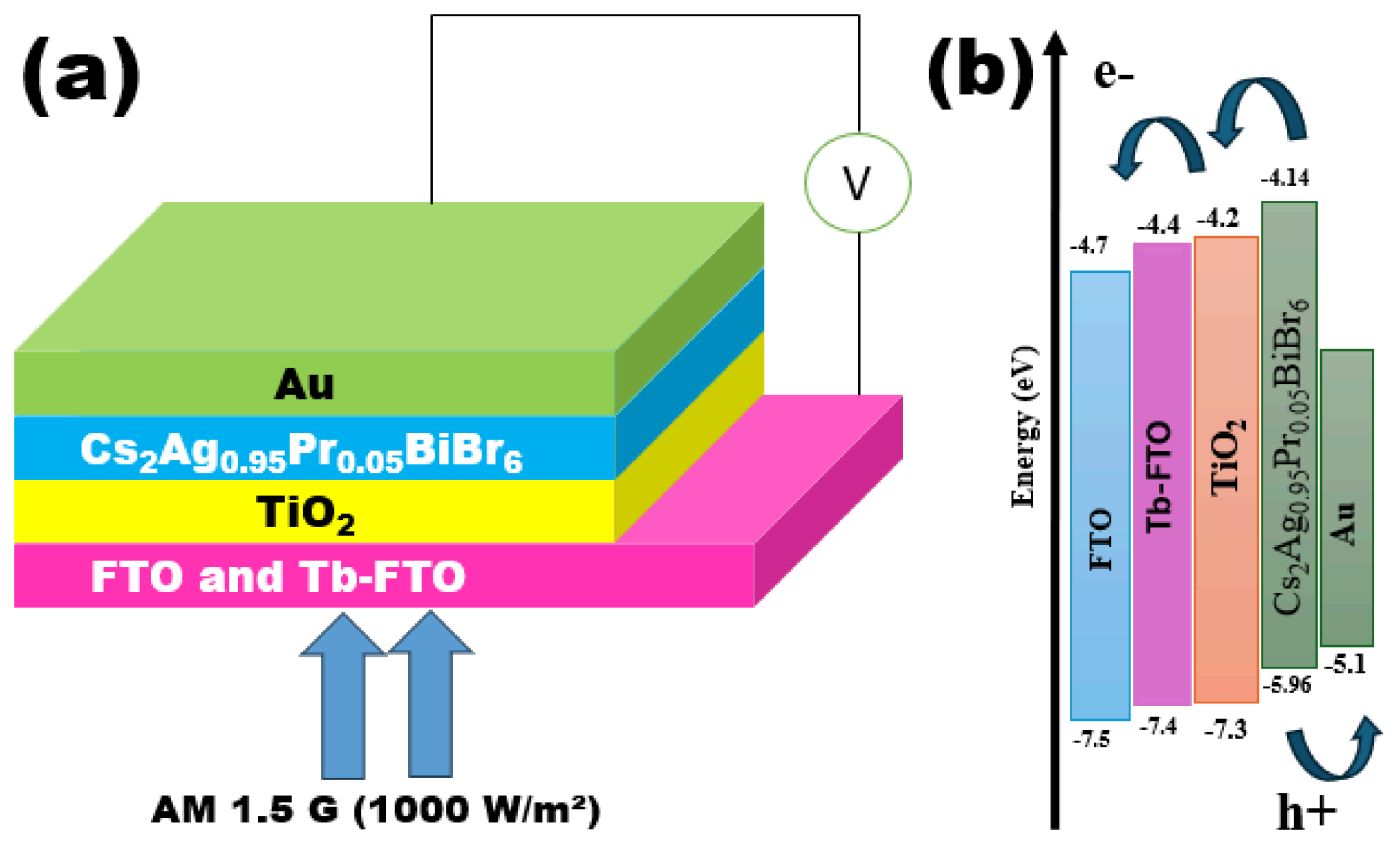
(**a**) Schematic of the device and (**b**) energy diagram.

Figure 11 depicts a device structure adapted from an earlier published design by Ullah et al. [2], but with a slight modification that involves the elimination of HTL to reduce degradation and suppress recombination. We considered the undoped FTO structure (FTO/TiO_2_/Cs_2_Ag_0.95_Pr_0.05_BiBr_6_/Au) and the doped FTO (Tb-FTO/TiO_2_/Cs_2_Ag_0.95_Pr_0.05_BiBr_6_/Au) designs. Light goes to the two devices through the fluorine tin oxide (FTO) and Tb-FTO contact. The absorber is Cs_2_Ag_0.95_Pr_0.05_BiBr_6_, and the electron transport layer is TiO_2_. The *W*_F_ of gold (Au), FTO and Tb-FTO are 5.1 eV, 4.4 eV, and 4.7 eV respectively [3,13].

## 4. Conclusions

A systematic investigation of the photovoltaic characteristics of Cs_2_Ag_0.95_Pr_0.05_BiBr_6_-based perovskite solar cells was carried out through SCAPS-1D numerical simulations. This investigation was conducted to evaluate the influence of rare-earth-metal doping on the front contact on photovoltaic performance. The undoped FTO-based PSC exhibited a *V*_oc_ of 0.86 V, *J*_sc_ of 12.52 mA/cm^2^, FF of 70.13%, and PCE of 7.51%. A comparable device architecture incorporating terbium-doped FTO was also simulated, yielding a *V*_oc_ of 0.86 V, a *J*_sc_ of 12.67 mA/cm^2^, an FF of 72.98%, and a PCE of 7.91%. Further enhancement was achieved through optimization of the absorber thickness and defect density, ETL thickness and doping level, absorber band gap, and ETL/absorber interfacial defect density. The Tb-FTO/TiO_2_/Cs_2_Ag_0.95_Pr_0.05_BiBr_6_/C device’s performance was optimized and achieved optimal values of 1.0 μm, 10^13^ cm^−2^, 0.09 μm, 10^21^ cm^−3^, 1.5 eV, and 10^8^ cm^−3^. The optimized device predicted a *V*_oc_ of 0.85 V, a *J*_sc_ of 27.73 mA/cm^2^, an FF of 84.51%, and a PCE of 19.93%. In addition, the effects of metal back contact *W*_F_, operating temperature, series and shunt resistances on device performance were systematically analyzed. The findings provide valuable insights that support the development of low-carbon and sustainable manufacturing pathways for perovskite-based energy technologies.

## Figures and Tables

**Table 2:** Impact of ETL/absorber defect density.

ETL/Absorber Defect Density (cm^−2^)	*V*_oc_ (V)	*J*_sc_ (mA/cm^2^)	FF (%)	PCE (%)
10^8^	0.86	12.68	72.79	7.96
10^9^	0.86	12.68	72.82	7.95
10^10^	0.86	12.67	72.98	7.91
10^11^	0.83	12.66	72.02	7.61
10^12^	0.81	12.50	65.07	6.59

**Table 3:** Impact of absorber defect density and ETL.

Band Gap (eV)	*V*_oc_ (V)	*J*_sc_ (mA/cm^2^)	FF (%)	PCE (%)
1.5	0.86	24.01	74.47	15.29
1.6	0.87	20.91	73.18	13.26
1.7	0.86	18.18	73.03	11.47
1.8	0.86	15.50	73.04	9.74
1.9	0.86	13.37	72.94	8.36
2.0	0.85	11.32	72.98	7.04
2.1	0.85	9.45	73.05	5.85

**Table 4:** Comparison of *J–V* characteristics from the current study and literature reports.

Device	Study	*V*_oc_ (V)	*J*_sc_ (mA/cm^2^)	FF (%)	PCE (%)	References
FTO/TiO_2_/Cs_1.96_li_0.01_Na_0.03_AgBiBr_6_/C	Exp	1.07	6.56	71.60	5.02	[19]
FTO/TiO_2_/Cs_2_Ag_0.95_GD_0.05_BiBr_6_/spiro-OMeTAD/au	Exp	0.91	5.58	75.00	3.74	[8]
FTO/Ti/ZTO-Cs_2_Ag_0.95_Mg_0.05_BiBr_6_/Au	Exp	0.91	5.40	81.10	3.98	[2]
FTO/TO_2_/Cs_2_AgBiBr_6_/C	Exp	0.43	9.4	38.00	1.63	[25]
ITO/SnO_2_/Cs_2_AgBiBr_6_/spiro-OMeTAD/Au	Exp	0.92	11.4	60.93	6.37	[20]
FTO/TiO_2_/Mo-Cs_2_AgBiBr_6_/Spiro-OMeTAD/Au	Exp.	0.94	5.59	67.00	3.95	[18]
FTO/TiO_2_/Cs_2_Ag_0.95_Pr_0.05_BiBr_6_/P3HT/Spiro-OMeTAD	Exp.	0.90	5.01	81.1	3.88	[2]
FTO/TiO_2_/IDL/Cs_2_AgBiBr_6_/C	Sim	1.69	12.11	88.79	18.21	[39]
ITO/SnO_2_/Cs_2_AgBiBr_6_/P3HT/Au	Sim	2.02	6.39	90.0	11.32	[40]
FTO/SnO_2_/Cs_2_AgBiBr_6_/Spiro-OMeTAD/Au	Sim	1.30	17.44	62.59	14.29	[41]
ITO/CdS/Cs_2_AgBiBr_6_/CuAlO_2_/Pt	Sim	1.64	4.91	88.74	7.16	[42]
FTO/TiO_2_/Cs_2_Ag_0.95_Pr_0.05_BiBr_6_/Au	sim	0.85	27.73	84.51	19.93	[This work]

**Table 5:** Variation in performance of *J–V* with different W_F_ of back contact.

Metal Contact	*W*_F_ (eV)	PCE (%)	*FF* (%)	*J*_sc_ (mA/cm^2^)	*V_oc_* (V)
Cr	4.5	4.52	64.54	27.66	0.25
Mo	4.62	7.51	72.68	27.69	0.37
Fe	4.81	12.37	79.22	27.71	0.56
C	5.0	17.35	83.09	27.73	0.75
Au	5.1	19.93	84.51	27.73	0.85
W	5.22	22.48	86.70	27.74	0.93
Ni	5.5	26.73	87.23	27.78	1.10
Pd	5.6	27.59	84.38	27.78	1.18
Pt	5.7	27.65	82.77	27.78	1.20
Se	5.9	27.65	82.69	27.78	1.20

**Table 6:** *PV* metrics for different temperatures.

Temperature (K)	PCE (%)	*FF* (%)	*J*_sc_ (mA/cm^2^)	*V_oc_* (V)
290	20.42	85.05	27.73	0.87
300	19.93	84.51	27.73	0.85
310	19.47	84.10	27.73	0.83
320	18.98	83.62	27.73	0.82
330	18.49	83.12	27.73	0.80

**Table 7:** *PV* metrics for varying Rs.

Rs (Ωcm^2^)	PCE (%)	*FF* (%)	*J*_sc_ (mA/cm^2^)	*V_oc_* (V)
0	19.93	84.51	27.73	0.85
2	18.55	78.63	27.73	0.85
4	17.18	72.79	27.72	0.85
6	15.80	66.96	27.72	0.85
8	14.45	61.25	27.72	0.85

**Table 8:** *PV* metrics for different Rsh.

Rsh (Ωcm^2^)	PCE (%)	*FF* (%)	*J*_sc_ (mA/cm^2^)	*V_oc_* (V)
10^2^	14.44	62.06	27.73	0.84
10^4^	19.87	84.28	27.73	0.85
10^6^	19.93	84.51	27.73	0.85
10^8^	19.93	84.51	27.73	0.85
10^10^	19.93	84.51	27.73	0.85

**Table 9:** Layer-specific data utilized for simulating the PSCs [2,12,13].

Parameters	FTO	Tb-FTO	TiO_2_	Cs_2_Ag_0.95_Pr_0.05_BiBr_6_
T (μm)	0.5	0.5	0.05	0.4
*E*_g_ (eV)	3.5	3.94	3.26	1.93
χ (eV)	4.3	4.3	4.2	3.88
*ϵ*_r_	9.0	9.0	10.0	5.8
*N*_C_ (cm^−3^)	2.2 × 10^18^	2.2 × 10^18^	2.2 × 10^18^	1.0 × 10^19^
*N*_V_ (cm^−3^)	1.8 × 10^19^	1.8 × 10^19^	1.8 × 10^19^	1.0 × 10^19^
μ_n_ (cm^2^ V^−1^ s^−1^)	20	30	20	11.81
μ_p_ (cm^2^ V^−1^ s^−1^)	10	15	10	0.49
$e\;v_{th}$ (cm/s)	1 × 10^7^	1 × 10^7^	1 × 10^7^	1 × 10^7^
$h\;v_{th}$ (cm/s)	1 × 10^7^	1 × 10^7^	1 × 10^7^	1 × 10^7^
*N*_D_ (cm^−3^)	1 × 10^18^	1 × 10^20^	1 × 10^17^	1 × 10^16^
*N*_A_ (cm^−3^)	0	0	0	1 × 10^16^
*N*_t_ (cm^−2^)	1 × 10^15^	1 × 10^15^	1 × 10^15^	1 × 10^15^

**Table 10:** Input data for modeling the interfacial layer.

Parameters	Absorber	ETL/Cs_2_Ag_0.95_Pr_0.05_BiBr_6_
Defect type	Neutral	Neutral
σ (e) (cm^2^)	1 × 10^−15^	2 × 10^−16^
σ (h) (cm^2^)	1 × 10^−15^	2 × 10^−16^
Energetic distribution	Gaussian	Single
Energy level with respect to Ev (eV)	0.600	0.600
Characteristic energy (eV)	0.1	0.1
*N*_t_ (cm^−2^)	1 × 10^15^	1 × 10^7^

## Data Availability

The data that support this paper will be made available upon reasonable request.
